# Homer1a Attenuates Hydrogen Peroxide-Induced Oxidative Damage in HT-22 Cells through AMPK-Dependent Autophagy

**DOI:** 10.3389/fnins.2018.00051

**Published:** 2018-02-09

**Authors:** Xiuquan Wu, Peng Luo, Wei Rao, Shuhui Dai, Lei Zhang, Wenke Ma, Jingnan Pu, Yang Yu, Jiu Wang, Zhou Fei

**Affiliations:** ^1^Department of Neurosurgery, Xijing Hospital, Fourth Military Medical University, Xi'an, China; ^2^Department of Neurosurgery, PLA Navy General Hospital, Beijing, China; ^3^Department of Neurosurgery, Baoji Center Hospital of Shanxi Province, Baoji, China

**Keywords:** Homer1a, hydrogen peroxide, AMPK, autophagy, oxidative stress, mitochondrial dysfunction

## Abstract

Neuronal oxidative stress is involved in diverse neurological disorders. Homer1a, as an important member of the Homer family and localized at the postsynaptic density, is known to protect cells against oxidative injury. However, the exact neuroprotective mechanism of Homer1a has not been fully elucidated. Here, we found that Homer1a promoted cell viability and reduced H_2_O_2_-induced LDH release. The overexpression of Homer1a enhanced autophagy after H_2_O_2_ treatment, which was confirmed by increased expression of LC3II, Beclin-1, and greater autophagosome formation. In addition, we demonstrated that activating autophagy improved cell survival and reduced H_2_O_2_-induced oxidative stress and mitochondrial damage. Moreover, the autophagy inhibitor 3-MA partially prevented the protective effects of Homer1a against oxidative challenge. We also found that the upregulation of Homer1a after H_2_O_2_ treatment increased the phosphorylation of AMPK. Furthermore, the AMPK inhibitor compound C inhibited Homer1a-induced autophagy and abolished Homer1a-mediated neuroprotection. All the above data suggests that Homer1a confers protection against H_2_O_2_-induced oxidative damage via AMPK-dependent autophagy.

## Introduction

Oxidative stress is an important pathophysiological feature of acute and chronic neurological diseases, including neurodegenerative diseases (Jiang et al., 2016), traumatic brain injury (Rodriguez-Rodriguez et al., 2014), and cerebral ischemia (Amaro et al., 2015). Oxidative stress occurs when the endogenous antioxidant capability of a cell is inadequate to overcome the effects of reactive oxygen species (ROS). H_2_O_2_, a well-established ROS generator, has been observed in nearly all types of oxidative stress, and oxygen radicals spread freely within and beyond cells and tissues (Barbouti et al., 2002). H_2_O_2_-induced oxidative stress can directly attack cellular components, such as lipids, proteins and DNA, and therefore leads to cell death.

Autophagy is primarily a protective process that occurs in response to various stresses. During the autophagy, double-membrane vesicles known as autophagosomes deliver cytosolic macromolecules and damaged organelles into the lysosome for degradation, thereby maintaining homeostasis (Bento et al., 2016). Although accumulating studies have highlighted the crosstalk between oxidative stress and autophagy, whether autophagy mitigates or exacerbates oxidative damage is still a controversial issue (Filomeni et al., 2015).

Homer proteins are a group of postsynaptic scaffold proteins that are characterized by a conserved enabled/vasodilator-stimulated phosphoprotein (Ena/VASP) homology 1 (EVH1) domain (Brakeman et al., 1997; Kato et al., 1997). Homer1a, as the short variant of Homer proteins, lacks the carboxy-terminal coiled-coil structure involved in the self-multimerization of long Homers (Shiraishi-Yamaguchi and Furuichi, 2007). Due to these structural features, Homer1a acts as a dominant-negative protein that disrupts the complexes formed by long Homers and regulates downstream signaling (Xiao et al., 2000). Our previous studies have demonstrated that Homer1a protects neurons against various stresses by regulating metabolic glutamate receptors, N-methyl-D-aspartate receptors, and store-operated calcium entry (Luo et al., 2014; Wang et al., 2015; Rao et al., 2016). We also found that Homer1a attenuates H_2_O_2_-induced oxidative stress by reducing ROS accumulation in PC12 cells (Luo et al., 2012). However, the exact associated molecular mechanisms of Homer1a against oxidative stress have not been reported. In the present study, we determined the protective effects of Homer1a against oxidative stress. We also investigated the effect of Homer1a overexpression on autophagy and confirmed the involvement of AMPK in autophagy and Homer1a-induced cytoprotection.

## Materials and methods

### Antibodies and reagents

Antibodies against LC3, P62, Beclin-1, β-actin, AMPK, and p-AMPK (Thr172), were obtained from Cell Signaling Technology. Antibodies against Homer1a were obtained from Synaptic Systems. Rapamycin, STF-62247, compound C, 3-MA, and chloroquine were purchased from Sigma-Aldrich.

### Cell culture

HT-22 cells (Institute of Biochemistry and Cell Biology, SIBS, CAS.) were grown in DMEM (Gibco, Frederick, MD, USA) plus 10% fetal bovine serum (Gibco) and 1% penicillin–streptomycin (Sigma-Aldrich, St. Louis, MO, USA). The cells were cultured in a humidified atmosphere at 37°C under 5% CO2. Before the experiments, HT-22 cells were seeded in 6-well culture dishes (10^6^ per well) and incubated until they reached 70–80% confluency. Rapamycin (5 μM), **STF-62247 (10** μ**M)**, 3-MA (2 mM), chloroquine (10 μM), and compound C (20 μM) were added to the cultures for 24 h before H_2_O_2_ treatment.

### Lentivirus construction and transfection

The preparation of lentivirus for the overexpression experiments was performed as previously described (Luo et al., 2014). The lentivirus overexpression system was developed by removing the EGFP open reading frame from the pGC-FU-EGFP-3FLAG construct (GeneChem Co., Shanghai, China) with an AgeI/NheI digestion and replacing this cassette with Homer1a cDNA.HT-22 cells were transfected with lentivirus vectors at a multiplicity of infection (MOI) of 30 for 48 h.

### Western blot

After each treatment, the cells were lysed in RIPA buffer with protease inhibitor cocktail (Roche Applied Bioscience, Indianapolis, IN, USA) and subjected to western blotting as described previously (Rao et al., 2016). Briefly, protein concentrations were assessed with a BCA Kit (Pierce, Rockford, IL, USA), and equivalent amounts of protein (30 μg) were separated by 8–15% SDS-PAGE gels, followed by transfer onto PVDF membranes. The membranes were then soaked in 5% skim milk in Tris-phosphate buffer containing 0.05% Tween 20 (TBST) for 1 h and further incubated overnight at 4°C with the appropriate primary antibody (Homer1a 1:1000; LC3II 1:1000; Beclin-1 1:1000; P62 1:8000; β-actin 1:5000; AMPK 1:1000; p-AMPK 1:1000). After three washes for 8 min in TBST, the blots were incubated with HRP-conjugated secondary antibodies for 1–2 h. The target protein signal was detected by SuperSignal West Pico Chemiluminescent Substrate (Thermos Scientific). The optical densities of the bands were quantified by ImageJ (Scion Corporation, Torrance, CA, USA).

### Measurement of ROS production

The intracellular ROS was measured by 2,7-dichlorodihydrofluoresceindiacetate (H_2_DCFDA) (Molecular Probe), as previously reported (Luo et al., 2012). HT-22 cells were incubated with H_2_DCFDA (10 mM) for 1 h at 37°C in dark and then resuspended in phosphate-buffered saline (PBS). The fluorescence intensity was read with a fluorescence plate reader (excitation wavelength of 480 nm and an emission wavelength of 530 nm).

### Measurement of lipid peroxidation

Malonyldialdehyde (MDA) and 4-hydroxynonenal (4-HNE), two indexes of lipid peroxidation, were detected using a Lipid Peroxidation 4-HNE Assay Kit (Beyotime, Shanghai, China) and a Lipid Peroxidation MDA Assay Kit (Beyotime, Shanghai, China), according to the manufacturer's instructions. The absorbance was read at 450 nm using a ELISA reader.

### Measurement of mitochondrial membrane potential (MMP)

MMP was monitored using the fluorescent dye rhodamine 123 (Rh 123). Rh 123 was added to cultures to achieve a final concentration of 10 mM for 30 min at 37°C after the HT-22 cells had been treated and washed with PBS. Fluorescence was acquired using a fluorescence plate reader (excitation wavelength of 480 nm, emission wavelength of 530 nm).

### Measurement of intracellular ATP

The intracellular ATP levels were measured using a firefly luciferase based ATP assay kit (Beyotime, China), strictly following the manufacturer's protocol. The ATP levels of each group were calculated as a percentage of the control.

### Immunofluorescence

HT-22 cells were fixed in 4% paraformaldehyde for 20 min, washed in PBS, and then permeabilized with 0.2% Triton X-100 for 10 min. Next, the cells were incubated with rabbit anti-LC3 antibody (1:200) at 4°C overnight, followed by incubation with Alexa 488 donkey-anti-rabbit IgG (1:400) for 2 h at room temperature. The nuclei were counter-stained with DAPI for 10 min (Sigma). All the images were acquired using a confocal microscope (FV10i, Olympus, Tokyo, Japan) with the same exposure time, light sensitivity and laser power.

### Cell viability assay

The cell viability assay was performed using the Cell Counting Kit-8 (CCK-8) (#CK04; Dojindo, Japan), following the manufacturer's instructions. Normal cells or lentivirus-infected cells were seeded in 96-well plates with 5,000 cells per well. After treatment, 10 μl/well of CCK-8 solution was added into each well and incubated for 4 h. The absorbance was measured at 450 nm using a microplate reader. (Bio-Rad, Hercules, CA, USA).

### Lactate dehydrogenase (LDH) assay

The release of LDH was evaluated using the Cytotoxicity Detection Kitplus (Roche Applied Bioscience, Indianapolis, IN, USA), following the manufacturer's protocol. After subtracting the background values in the medium, the percentage cytotoxicity was calculated with the following equation: LDH release (% of Max) = 100 × (experimental value–low control)/(high control–low control). Experimental value, LDH values in the experimental groups; Low control, LDH values in the untreated normal cells; High control, the maximum releasable LDH values in the untreated normal cells.

### Tunel staining

Apoptosis in HT-22 cells was detected with the terminal deoxynucleotidyl transferase (TdT)-mediated dUTP nick-end labeling (TUNEL) In Situ Cell Death Detection Kit (#11684795910; Roche, Mannheim, Germany). The cells were fixed with freshly prepared 4% paraformaldehyde for 20 min at room temperature and permeabilized with 0.2% Triton X-100 for 5 min. The cells were then incubated with 50 μl TUNEL reagent mixture for 60 min at 37°C following the manufacturer's protocol. The images were captured with a fluorescence microscope, the TUNEL-positive cells were counted and the ratio of TUNEL-positive cells/total cells was calculated. DAPI (10 μg/ml) was used to stain nuclei.

### Electron microscopy

The HT-22 cells were fixed in 2% paraformaldehyde-2% glutaraldehyde buffered with 0.1 mol/L phosphate buffer at room temperature. The cells were post-fixed with 1% osmium tetroxide, dehydrated in acetone and immersed in resin. After hardening, the samples were cut into 50 nm thick slices, stained with lead citrate and observed on an electron microscope.

### Statistical analysis

All the experiments were performed a minimum of three times. The statistical analyses were conducted using the GraphPad Prism software, version 6.0 (GraphPad, San Diego, CA, USA). Significance between experiments was assessed by univariate analysis of variance (ANOVA; more than two groups), followed by Bonferroni's multiple comparisons or unpaired *t*-test (two groups).

## Results

### Homer1a protects against H_2_O_2_-induced oxidative injury in HT-22 cells

**Figure 1:**
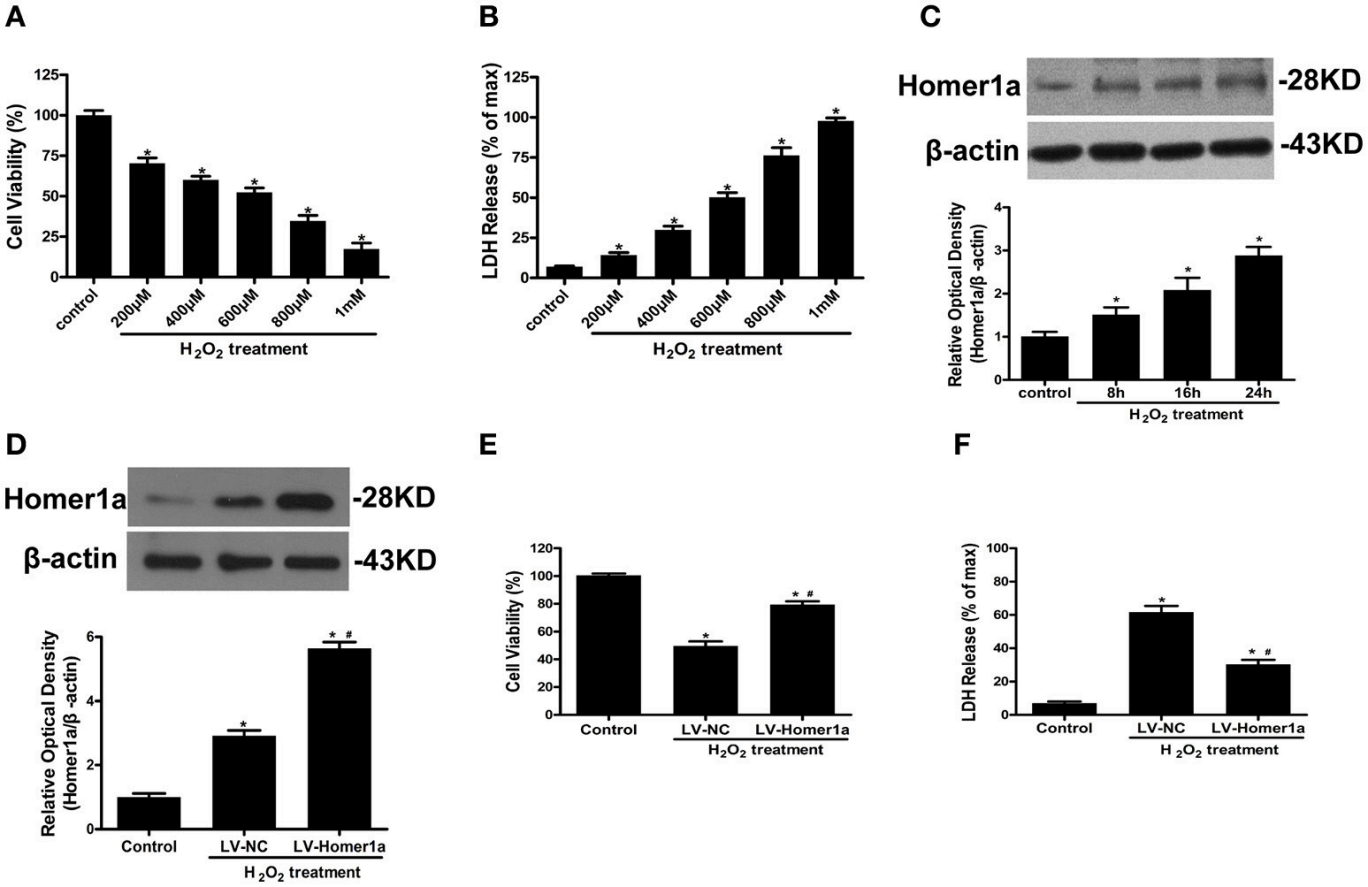
Homer1a protects against H_2_O_2_-induced oxidative injury in HT-22 cells. HT-22 cells were exposed to different concentrations of H_2_O_2_ for 24 h. Cell viability was measured by CCK-8 assay **(A)**, and cytotoxicity was detected by LDH release assay **(B)**. HT-22 cells were treated with 600 μM H_2_O_2_ for indicated times, the expression of Homer1a was determined by Western blot **(C)**. HT-22 cells were transfected with LV-NC or LV-Homer1a for 48 h and exposed to H_2_O_2_ (600 μM) for 24 h. Homer1a protein level was detected by Western blot after treated with or without H_2_O_2_ **(D)**. Cell viability and cytotoxicity were measured in presence or absence of H_2_O_2_ **(E,F)**. The data were expressed as means ± SEM from five experiments. ^*^P < 0.05 vs. Control, ^#^*P* < 0.05 vs. LV-NC group.

HT-22 cells were exposed to an increasing concentration of H_2_O_2_ (200, 400, 600, 800, 1000 μM) for 24 h. The results showed that H_2_O_2_ decreased cell viability and induced LDH release in a dose-dependent manner (Figures 1A,B). An exposure to 600 μM H_2_O_2_ for 24 h was used in the following experiments given that exposure to the cell insult induced nearly 50% cell death. To investigate the effect of H_2_O_2_ on Homer1a expression, HT-22 cells were incubated in the presence of H_2_O_2_ (600 μM) for different periods of time (control, 8, 16, and 24 h). The results indicated that H_2_O_2_ significantly increased the levels of Homer1a within 24 h (Figure 1C).

To identify the effect of Homer1a on H_2_O_2_-induced oxidative stress, HT-22 cells were transfected with lentivirus carrying Homer1a (LV-Homer1a) or a negative control lentivirus (LV-NC). Immunoblot analysis showed that lentiviral transduction of LV-Homer1a increased the expression of Homer1a protein (Figure 1D). After treatment with H_2_O_2_ for 24 h, the viability of HT-22 cells transfected with LV-Homer1a was higher than the cells transfected with LV-NC (Figure 1E). Furthermore, the overexpression of Homer1a clearly decreased LDH release after H_2_O_2_ treatment (Figure 1F).

### Homer1a modulates autophagy in HT-22 cells undergoing oxidative stress

**Figure 2:**
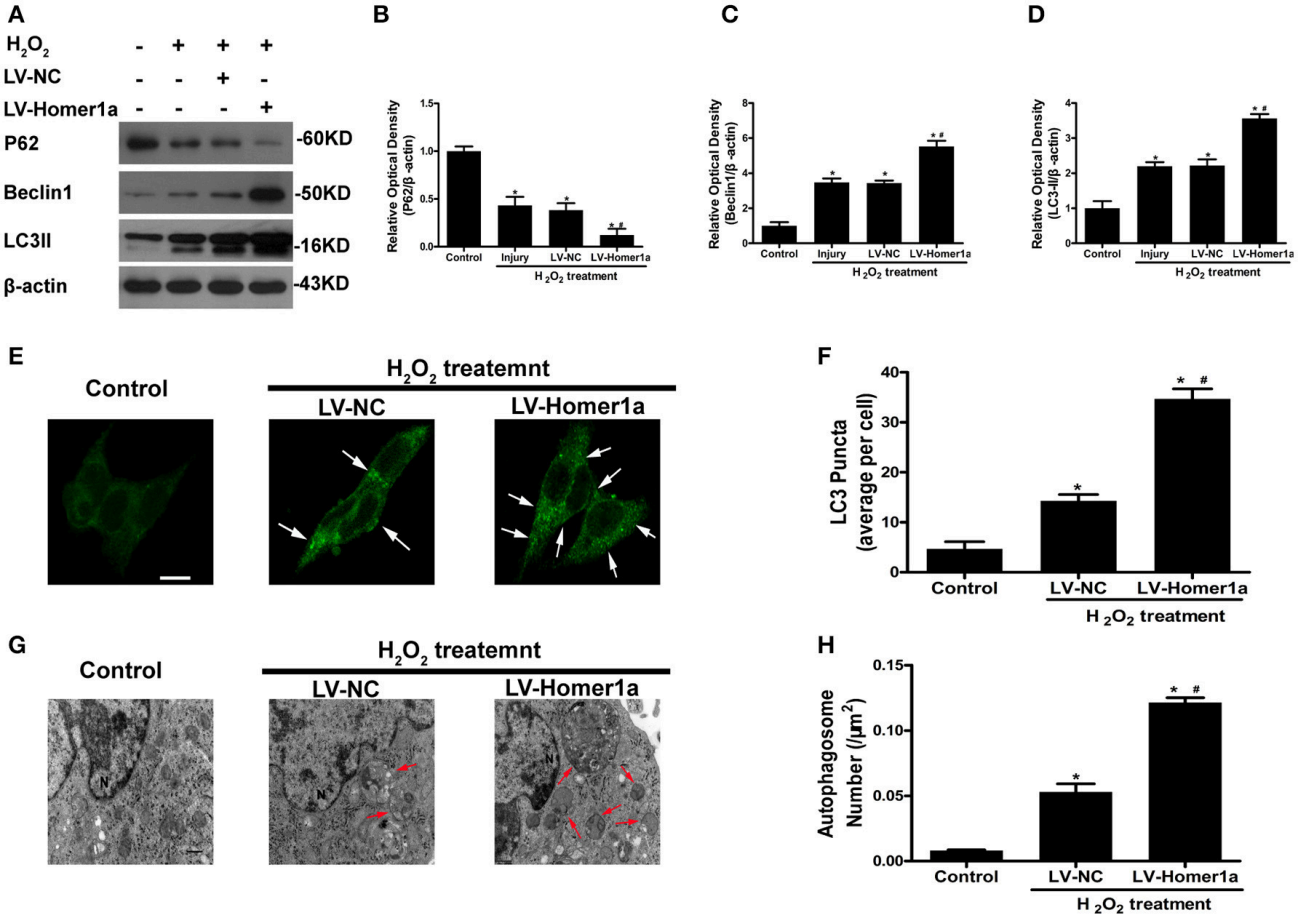
Homer1a regulates autophagy following oxidative stress. HT-22 cells were transfected with LV-NC or LV-Homer1a for 48 h and exposed to H_2_O_2_ (600 μM) for 24 h. The expression of LC3II, Beclin1 and P62 at 24 h after H_2_O_2_ treatment were detected by Western blot analysis **(A–D)**. LC3II was also detected with Immunofluorescence staining at 24 h after H_2_O_2_ treatment **(E)**, and the number of LC3 puncta (arrows) were calculated **(F)**. Scale bar = 10 μm. HT-22 cells were observed by electron microscopy at 24 h after H_2_O_2_ treatment **(G)**, and the number of autophagosomes (red arrows) were calculated **(H)**. Scale bar = 500 nm_._ N: nucleus. The data were expressed as means ± SEM from five experiments. ^*^P < 0.05 vs. Control, ^#^*P* < 0.05 vs. LV-NC group.

To test whether the Homer1a regulates autophagy following oxidative stress, we transfected HT-22 cells with LV-Homer1a and cultured the cells for 2 days before adding H_2_O_2_. Our results showed that the overexpression of Homer1a increased protein levels of LC3II and Beclin-1 and decreased the expression of p62 (Figures 2A–D). The immunofluorescent results indicated that the overexpression of Homer1a significantly increased the number of LC3-positive puncta after H_2_O_2_ treatment compared to the LV-NC group (Figures 2E,F). In addition, ultrastructural studies clearly revealed more autophagosomes in the LV-Homer1a group after H_2_O_2_ treatment compared to the LV-NC group (Figures 2G,H).

### Homer1a inhibits H_2_O_2_-induced cell injury by upregulating autophagy

**Figure 3:**
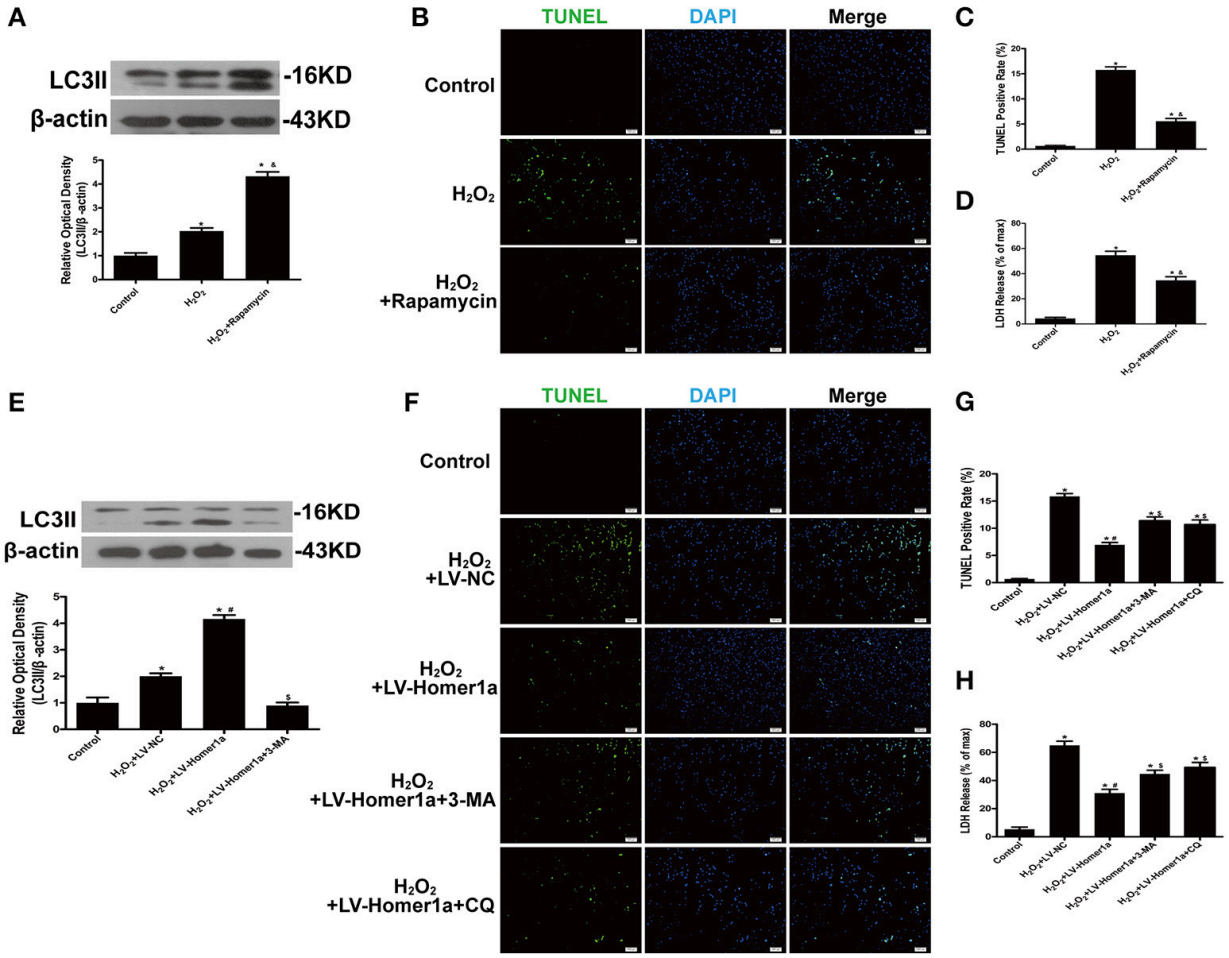
Homer1a inhibits H_2_O_2_-induced cell injury by upregulating autophagy. HT-22 cells were treated with rapamycin (5 μM) for 24 h then H_2_O_2_ applied for 24 h. The expression of LC3II was determined by Western blot **(A)**. Scale bar = 100 μm. Apoptotic cell death was measured by TUNEL staining **(B,C)**, and cytotoxicity was detected by LDH release assay **(D)**. HT-22 cells were transfected with LV-NC or LV-Homer1a for 48 h. After transfection, the cells were treated with 3-MA (2 mM) or CQ (10 μM) for 24 h then H_2_O_2_ applied for 24 h. The expression of LC3II was determined by Western blot **(E)**. Scale bar = 100 μm. Apoptotic cell death was measured by TUNEL staining **(F,G)**, and cytotoxicity was detected by LDH release assay **(H)**. The data were expressed as means ± SEM from five experiments. ^*^P < 0.05 vs. Control, ^&^*P* < 0.05 vs. H_2_O_2_, ^#^*P* < 0.05 vs. H_2_O_2_+LV-NC, ^$^*P* < 0.05 vs. H_2_O_2_+LV-Homer1a.

To determine the role of autophagy in H_2_O_2_-induced oxidative damage, we evaluated cell injury after HT-22 cells were treated with H_2_O_2_ and pharmacological agents that modulated autophagy (Figure 3A). The results indicated that rapamycin, a classical inducer of autophagy, reduced the number of TUNEL-positive cells and decreased LDH release after oxidative stress (Figures 3B–D). To identify whether Homer1a conferred protection through modulation of autophagy, HT-22 cells were transfected with LV-Homer1a and/or treated with the autophagy inhibitor 3-MA. The results showed that the increased expression of LC3II induced by Homer1a overexpression was decreased by 3-MA (Figure 3E). Moreover, treatment with 3-MA or chloroquine (CQ), another autophagy inhibitor, partially reversed the protective effects of Homer1a against H_2_O_2_-induced injury (Figures 3F–H).

### Autophagy is involved in the homer1a-mediated protection against H_2_O_2_-induced oxidative stress and mitochondrial damage

**Figure 4:**
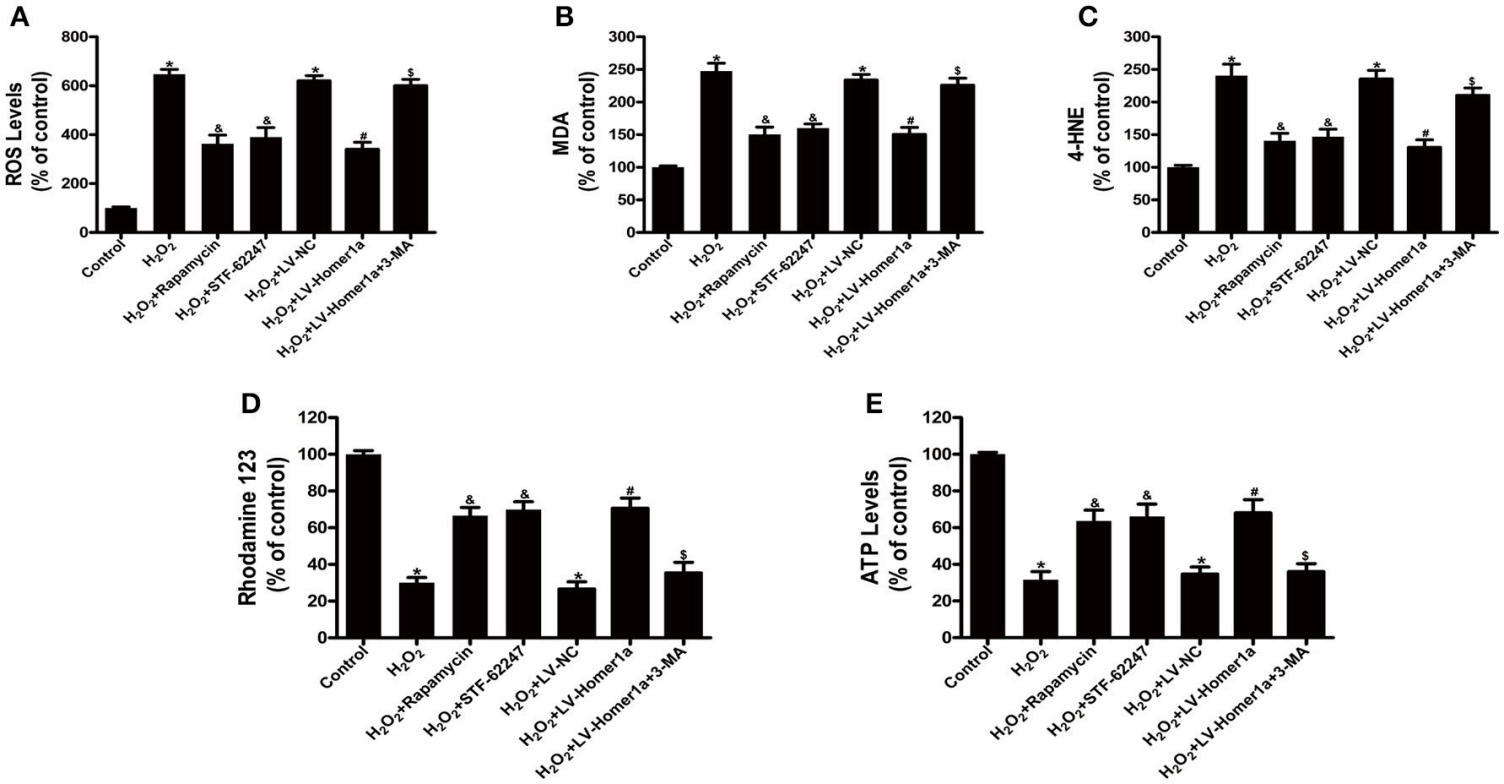
Autophagy is involved in the Homer1a-mediated protection against H_2_O_2_-induced oxidative stress and mitochondrial damage. HT-22 cells were treated with rapamycin (5 μM) or STF-62247 (10 μM) for 24 h then H_2_O_2_ applied for 24 h. ROS production, lipid peroxidation, MMP and ATP were assayed **(A–E)**. HT-22 cells were transfected with LV-NC or LV-Homer1a for 48 h. After transfection, the cells were treated with 3-MA (2 mM) for 24 h then H_2_O_2_ applied for 24 h. ROS production, lipid peroxidation, MMP and ATP were assayed **(A–E)**. The data were expressed as means ± SEM from five experiments. ^*^P < 0.05 vs. Control, ^&^*P* < 0.05 vs. H_2_O_2_, ^#^*P* < 0.05 vs. H_2_O_2_+LV-NC, ^$^*P* < 0.05 vs. H_2_O_2_+LV-Homer1a.

To assess the relationship between autophagy, H_2_O_2_-induced oxidative stress and mitochondrial damage, HT-22 cells were pretreated with rapamycin or STF-62247, another autophagy activator before H_2_O_2_ treatment. The results showed that rapamycin and STF-62247 both significantly reduced H_2_O_2_-induced ROS production, lipid peroxidation (MDA and 4-HNE), loss of MMP and ATP production (Figures 4A–E). To further investigate the role of Homer1a-induced autophagy in regulating oxidative stress and mitochondrial function, HT-22 cells were transfected with LV-Homer1a or LV-NC and treated with 3-MA and H_2_O_2_. We observed that H_2_O_2_-induced ROS production and lipid peroxidation decreased in HT-22 cells transfected LV-Homer1a (Figures 4A–C). Moreover, the overexpression of Homer1a prevented the H_2_O_2_-induced loss of MMP and reduction of ATP production (Figures 4D,E). However, the Homer1a-mediated protection against H_2_O_2_-induced oxidative stress and mitochondrial damage were partially abolished by 3-MA (Figures 4A–E).

### The homer1a/AMPK/autophagy pathway reduces H_2_O_2_-induced oxidative stress

**Figure 5:**
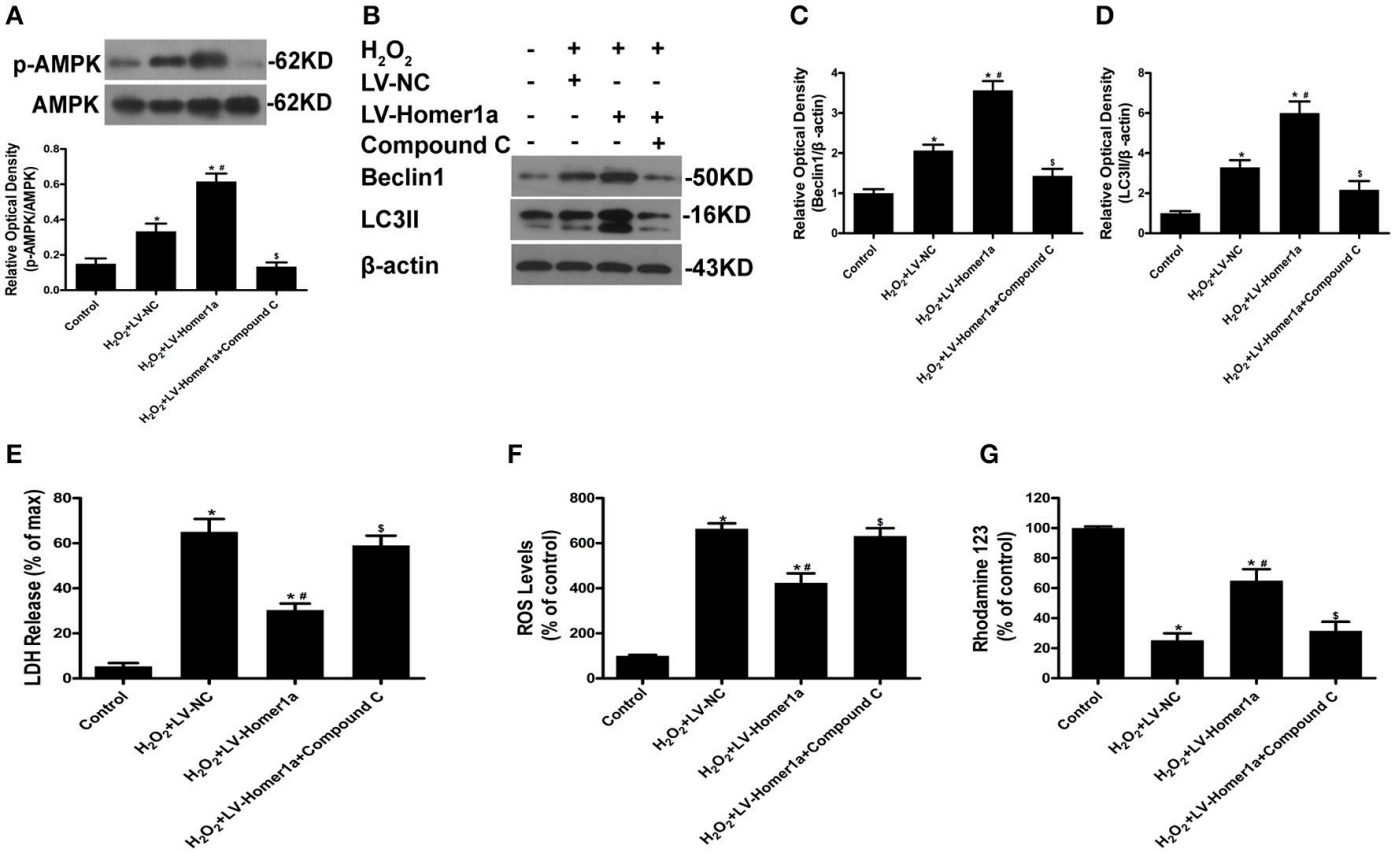
The Homer1a/AMPK/autophagy pathway reduces H_2_O_2_-induced oxidative stress. HT-22 cells were transfected with LV-NC or LV-Homer1a for 48 h. After transfection, the cells were treated with compound C (20 μM) for 24 h then H_2_O_2_ applied for 24 h. The expression of p-AMPK, AMPK, Beclin1 and LC3II were detected by Western blot analysis **(A–D)**. Cytotoxicity was detected by LDH release assay **(E)**. ROS production and MMP were assayed **(F,G)**. The data were expressed as means ± SEM from five experiments. ^*^P < 0.05 vs. Control, ^#^*P* < 0.05 vs. H_2_O_2_+LV-NC, ^$^*P* < 0.05 vs. H_2_O_2_+LV-Homer1a.

To further clarify the molecular mechanisms through which Homer1a mediates autophagy and protection, we tested whether Homer1a protects HT-22 cells through the AMPK pathway. The results indicated that the overexpression of Homer1a markedly increased the phosphorylation of AMPK after H_2_O_2_ treatment (Figure 5A). In addition, the increased levels of p-AMPK induced by Homer1a was partially prevented by the AMPK inhibitor compound C (Figure 5A). The western blot results showed that the increased expression of Beclin-1 and LC3II induced by Homer1a overexpression after oxidative stress was reversed by compound C treatment (Figures 5B–D). Moreover, the Homer1a-induced decreased LDH release, ROS production and increase in MMP levels upon H_2_O_2_ challenge were partially abolished by compound C, suggesting that AMPK was involved in Homer1a-induced protection (Figures 5E–G).

## Discussion

In the present study, we revealed that Homer1a confers protection against oxidative stress by regulating autophagy in HT-22 cells. First, we observed that the overexpression of Homer1a attenuated H_2_O_2_-induced oxidative injury. Second, it was determined that the protective functions of Homer1a were associated with activation of autophagy. Lastly, the AMPK pathway was responsible for the regulation of autophagy induced by Homer1a.

Homer1a, as an immediate early gene (IEG), functions directly at the synapses (Serchov et al., 2016). In general, Homer1a appears to be an important regulator of activity-induced remodeling at synaptic structures (Inoue et al., 2007). Previous studies have shown that activation of Homer1a improved neuronal survival after acute brain injury, such as traumatic neuronal injury, cerebral ischemia, and excitotoxic challenge (Luo et al., 2014; Fei et al., 2015; Wang et al., 2015). In line with these studies, our results indicated that the levels of Homer1a protein in HT-22 cells increased after H_2_O_2_ treatment in a time-dependent manner. The overexpression of Homer1a significantly increased cell viability and decreased LDH release after oxidative stress, which strongly suggested that Homer1a might be cytoprotective against oxidative stress.

Autophagy is a catabolic process occurring in response to multiple forms of cellular stress, such as nutrient deprivation, hypoxia, and intracellular pathogens (Kroemer et al., 2010). It is well accepted that oxidative stress, as the converging point of these stimuli, is the primary intracellular signal transducer that sustains autophagy (Filomeni et al., 2015). Intriguingly, enhanced autophagy is considered both a type of cell death and a pro-survival mechanism upon oxidative stress. In our *in vitro* model, autophagy activitors, rapamycin and STF-62247 both reduced ROS production, lipid peroxidation, and mitochondrial dysfunction after H_2_O_2_ treatment. These results were consistent with a previous study showing that the upregulation of autophagy with rapamycin in differentiated, rotenone-treated SH-SY5Y cells preserved cell viability (Pan et al., 2009). These results indicated that autophagy is beneficial for cells against oxidative stress. Conversely, recent studies found that treatment with 3-MA protected HT-22 cells against glutamate-induced oxidative stress (Yang et al., 2017). The inconsistency between our results and previous studies might partially be attributed to different models and time points.

According to our results, Homer1a is an important regulator of autophagy under oxidative stress conditions. Our previous study indicated that Homer1a protected against NMDA-induced neuronal injury by disassembling NR2B-PSD95-nNOS complexes and reducing the membrane distribution of NMDARs (Wang et al., 2015). Importantly, it is well accepted that autophagy machinery is robustly induced in cultured neurons subjected to NMDA treatment (Sadasivan et al., 2010). Therefore, the relationship between Homer1a and autophagy is deserving of attention. Our present results suggest that Homer1a overexpression markedly increased the expression of LC3II and Beclin1, as well as LC3-positive puncta and the number of autophagosomes after H_2_O_2_ treatment. Moreover, our previous study proved that the overexpression of Homer1a protected against H_2_O_2_-induced oxidative stress by reducing ROS production and the loss of MMP and ATP production (Luo et al., 2012). However, we found that autophagy inhibitor 3-MA abrogated the Homer1a-mediated protection against H_2_O_2_-induced oxidative stress and mitochondrial damage. Taken together, our results strongly demonstrated that Homer1a protected against H_2_O_2_-induced oxidative injury by inducing autophagy.

Another issue to be considered is how Homer1a interacts with the autophagy pathway. We found that the overexpression of Homer1a increased the phosphorylation of AMPK, which can trigger autophagy to coordinate cell growth and metabolism (Mihaylova and Shaw, 2011). Moreover, AMPK has a pivotal role in the interplay between oxidative stress and autophagic machinery (Filomeni et al., 2015). Previous studies have shown that Homer1a can attenuate oxidative injury through store-operated calcium entry (SOCE), which can also be regulated by AMPK (Lang et al., 2013; Rao et al., 2016). Intriguingly, our results showed that the Homer1a-induced increase in p-AMPK levels was partially reversed by treatment with compound C. Accordingly, the enhanced autophagy and neuroprotection induced by Homer1a were abolished by compound C. These data indicate that Homer1a stimulated the activity of AMPK and increased the autophagy rate, thereby attenuating H_2_O_2_-induced injury.

In conclusion, oxidative stress-induced cell damage is common in the context of neurological diseases. According to our results, Homer1a can protect against oxidative stress through AMPK-dependent autophagy activation, providing a new perspective on the protective role of Homer1a. However, little is known regarding the effect of Homer1a on autophagy flux and mitophagy. Further investigations on the link between Homer1a and autophagy signaling would provide a better understanding of neurological diseases.

## Author contributions

XW and ZF: designed the study; XW, PL, WR, and SD: performed the experiments; LZ, WM, and JP: analyzed the data and prepared the figures; YY and JW: contributed reagents or materials; XW and PL: wrote the manuscript; ZF: helped to revise the manuscript.

### Conflict of interest statement

The authors declare that the research was conducted in the absence of any commercial or financial relationships that could be construed as a potential conflict of interest.

## References

[B1] Amaro S. Llull L. Renú A. Laredo C. Perez B. Vila E.. (2015). Uric acid improves glucose-driven oxidative stress in human ischemic stroke. Ann. Neurol. 77, 775–783. 10.1002/ana.24378 25627874

[B2] Barbouti A. Doulias P. T. Nousis L. Tenopoulou M. Galaris D. (2002). DNA damage and apoptosis in hydrogen peroxide-exposed Jurkat cells: bolus addition versus continuous generation of H_(2)_O_(2)_. Free Radic. Biol. Med. 33, 691–702. 10.1016/S0891-5849(02)00967-X 12208356

[B3] Bento C. F. Renna M. Ghislat G. Puri C. Ashkenazi A. Vicinanza M.. (2016). Mammalian autophagy: how does it work? Annu. Rev. Biochem. 85, 685–713. 10.1146/annurev-biochem-060815-014556 26865532

[B4] Brakeman P. R. Lanahan A. A. O'Brien R. Roche K. Barnes C. A. Huganir R. L.. (1997). Homer: a protein that selectively binds metabotropic glutamate receptors. Nature 386, 284–288. 9069287 10.1038/386284a0

[B5] Fei F. Li J. Rao W. Liu W. Chen X. Su N.. (2015). Upregulation of Homer1a promoted retinal ganglion cell survival after retinal ischemia and reperfusion via interacting with Erk pathway. Cell. Mol. Neurobiol. 35, 1039–1048. 10.1007/s10571-015-0198-2 25924704 PMC11486256

[B6] Filomeni G. De Zio D. Cecconi F. (2015). Oxidative stress and autophagy: the clash between damage and metabolic needs. Cell Death Differ. 22, 377–388. 10.1038/cdd.2014.150 25257172 PMC4326572

[B7] Inoue Y. Udo H. Inokuchi K. Sugiyama H. (2007). Homer1a regulates the activity-induced remodeling of synaptic structures in cultured hippocampal neurons. Neuroscience 150, 841–852. 10.1016/j.neuroscience.2007.09.081 18006237

[B8] Jiang T. Sun Q. Chen S. (2016). Oxidative stress: a major pathogenesis and potential therapeutic target of antioxidative agents in Parkinson's disease and Alzheimer's disease. Prog. Neurobiol. 147, 1–19. 10.1016/j.pneurobio.2016.07.005 27769868

[B9] Kato A. Ozawa F. Saitoh Y. Hirai K. Inokuchi K. (1997). vesl, a gene encoding VASP/Ena family related protein, is upregulated during seizure, long-term potentiation and synaptogenesis. FEBS Lett. 412, 183–189. 10.1016/S0014-5793(97)00775-8 9257717

[B10] Kroemer G. Mariño G. Levine B. (2010). Autophagy and the integrated stress response. Mol. Cell 40, 280–293. 10.1016/j.molcel.2010.09.023 20965422 PMC3127250

[B11] Lang F. Münzer P. Gawaz M. Borst O. (2013). Regulation of STIM1/Orai1-dependent Ca2+ signalling in platelets. Thromb Haemost. 110, 925–930. 10.1160/TH13-02-0176 23846758

[B12] Luo P. Chen T. Zhao Y. Xu H. Huo K. Zhao M.. (2012). Protective effect of Homer 1a against hydrogen peroxide-induced oxidative stress in PC12 cells. Free Radical Res. 46, 766–776. 10.3109/10715762.2012.678340 22435683

[B13] Luo P. Chen T. Zhao Y. Zhang L. Yang Y. Liu W.. (2014). Postsynaptic scaffold protein Homer 1a protects against traumatic brain injury via regulating group I metabotropic glutamate receptors. Cell Death Dis. 5:e1174. 10.1038/cddis.2014.116 24722299 PMC5424101

[B14] Mihaylova M. M. Shaw R. J. (2011). The AMPK signalling pathway coordinates cell growth, autophagy and metabolism. Nat. Cell Biol. 13, 1016–1023. 10.1038/ncb2329 21892142 PMC3249400

[B15] Pan T. Rawal P. Wu Y. Xie W. Jankovic J. Le W. (2009). Rapamycin protects against rotenone-induced apoptosis through autophagy induction. Neuroscience 164, 541–551. 10.1016/j.neuroscience.2009.08.014 19682553

[B16] Rao W. Peng C. Zhang L. Su N. Wang K. Hui H.. (2016). Homer1a attenuates glutamate-induced oxidative injury in HT-22 cells through regulation of store-operated calcium entry. Sci. Rep. 6:33975. 10.1038/srep33975 27681296 PMC5041114

[B17] Rodriguez-Rodriguez A. Egea-Guerrero J. J. Murillo-Cabezas F. Carrillo-Vico A. (2014). Oxidative stress in traumatic brain injury. Curr. Med. Chem. 21, 1201–1211. 10.2174/0929867321666131217153310 24350853

[B18] Sadasivan S. Zhang Z. Larner S. F. Liu M. C. Zheng W. Kobeissy F. H.. (2010). Acute NMDA toxicity in cultured rat cerebellar granule neurons is accompanied by autophagy induction and late onset autophagic cell death phenotype. BMC Neurosci. 11:21. 10.1186/1471-2202-11-21 20167092 PMC2836363

[B19] Serchov T. Heumann R. van Calker D. Biber K. (2016). Signaling pathways regulating Homer1a expression: implications for antidepressant therapy. Biol. Chem. 397, 207–214. 10.1515/hsz-2015-0267 26641965

[B20] Shiraishi-Yamaguchi Y. Furuichi T. (2007). The Homer family proteins. Genome Biol. 8:206. 10.1186/gb-2007-8-2-206 17316461 PMC1852408

[B21] Wang Y. Rao W. Zhang C. Zhang C. Liu M. D. Han F.. (2015). Scaffolding protein Homer1a protects against NMDA-induced neuronal injury. Cell Death Dis. 6:e1843. 10.1038/cddis.2015.216 26247728 PMC4558508

[B22] Xiao B. Tu J. C. Worley P. F. (2000). Homer: a link between neural activity and glutamate receptor function. Curr. Opin. Neurobiol. 10, 370–374. 10.1016/S0959-4388(00)00087-8 10851183

[B23] Yang Y. Luo P. Xu H. Dai S. Rao W. Peng C.. (2017). RNF146 inhibits excessive autophagy by modulating the Wnt-β-Catenin pathway in glutamate excitotoxicity injury. Front. Cell Neurosci. 11:59. 10.3389/fncel.2017.00059 28321181 PMC5337692

